# Coverage, reporting degree and design of the Swedish quality registry for patients born with cleft lip and/or palate

**DOI:** 10.1186/s12913-020-05389-x

**Published:** 2020-06-11

**Authors:** Kristina Klintö, Agneta Karsten, Agneta Marcusson, Anna Paganini, Sara Rizell, Jenny Cajander, Karin Brunnegård, Malin Hakelius, Åsa Okhiria, Petra Peterson, Avni Abdiu, Christina Havstam, Hans Mark, Emilie Hagberg, Lena Björnström, Anna-Paulina Wiedel, Magnus Becker

**Affiliations:** 1 Department of Clinical Sciences in Malmö, Lund University, Malmö, Sweden; 2 Department of Otorhinolaryngology, Division of Speech and Language Pathology, Skåne University Hospital, Jan Waldenströms gata 18, S-205 02 Malmö, Sweden; 3 Division of Orthodontics, Department of Dental Medicine, Karolinska Institutet, and Stockholm Craniofacial Team, Karolinska Institutet, and Stockholm Craniofacial Team, Karolinska University Hospital, Stockholm, Sweden; 4 Maxillofacial Unit, and Department of Clinical and Experimental Medicine, Linköping University, Linköping, Sweden; 5 Department of Plastic Surgery, Sahlgrenska University Hospital, Gothenburg, Sweden; 6 Orthodontic Clinic, University Clinics of Odontology and Gothenburg University, Gothenburg, Sweden; 7 Department for Surgical and Perioperative Sciences, Umeå University, Umeå, Sweden; 8 Speech and Language Therapy Unit, University Hospital of Umeå, Umeå, Sweden; 9 Department of Plastic and Maxillofacial Surgery, Uppsala University Hospital, Uppsala, Sweden; 10 Department of Speech-Language Pathology, Uppsala University Hospital, Uppsala, Sweden; 11 Stockholm Craniofacial Team, Department of Craniofacial Diseases, Karolinska University Hospital, Stockholm, Sweden; 12 Department of Hand Surgery, Plastic Surgery, and Burns, and Department of Clinical and Experimental Medicine, Linköping University, Linköping, Sweden; 13 Department of Otorhinolaryngology, Division of Speech and Language Pathology, Sahlgrenska University Hospital, Gothenburg, Sweden; 14 Functional Area Speech and Language Pathology and Stockholm Craniofacial Team, Karolinska University Hospital, Stockholm, Sweden; 15 Specialized Dental Care, Orthodontics, County Council of Västerbotten, Umeå, Sweden; 16 Department of Maxillofacial Surgery, Skåne University Hospital, Malmö, Sweden; 17 Department of Plastic Surgery, Skåne University Hospital, Malmö, Sweden

**Keywords:** Cleft lip, Cleft palate, Registry, Surgery, Orthodontics, Speech

## Abstract

**Background:**

The objective of the Swedish cleft lip and palate (CLP) registry is to promote quality control, research and improvement of treatment, by comparison of the long-term results of surgery, orthodontics and speech from all six Swedish CLP centres. The purpose of the study was to investigate the coverage and reporting degree of the Swedish CLP registry, and to describe the design of the registry and discuss questions of reliability and validity of the data included.

**Methods:**

All six Swedish CLP centres participate in the registry. All children in Sweden with cleft lip and/or cleft palate, born from 2009 onwards, are included in the registry. Baseline data such as cleft type (ICD-10 diagnosis), heredity, birth weight and additional deformities and/or syndromes, as well as pre-surgical treatment, are recorded at first visit. Data on surgical treatment are recorded continuously. Treatment outcome regarding dentofacial development and speech are recorded at follow-ups at 5, 10, 16 and 19 years of age. Data on dentofacial development are also recorded 1 year after orthognathic surgery. In addition, data on babbling and speech are recorded at 18 months of age. Coverage degree and reporting degree of surgery was assessed by comparison with registrations in the Swedish Central patient registry. Reporting degree of orthodontic and speech registrations at 5 years of age was assessed by comparison with registrations at baseline.

**Results:**

The average coverage degree for children born 2009 to 2018 was 95.1%. For cleft-related surgeries, the average reporting degree was 92.4%. Average reporting degree of orthodontic registrations and speech registrations at age 5 years was 92 and 97.5% respectively.

**Conclusion:**

In order to achieve valid and reliable data in a healthcare quality registry, the degree of coverage and reporting needs to be high, the variables included should be limited and checked for reliability, and the professionals must calibrate themselves regularly. The Swedish CLP registry fulfils these requirements.

## Background

Around 1/500 Swedish children are born with cleft lip with or without cleft palate (CL/P) [1]. Additionally, internationally adopted children and immigrants have increased the prevalence of CL/P in Sweden. Children are treated at one of the six Swedish regional cleft lip and palate (CLP) centres depending on where they are born and are followed up to the age of 19 years. All six CLP centres are connected to the Swedish quality registry for patients with CL/P (CLP registry). The surgical methods for palatoplasty, and time for palatal repair, vary among the centres. Currently in Sweden, the children are treated with a primary lip plasty with simultaneous correction of the nasal cartilages at 3 to 6 months of age. The palate is either closed in one stage at 9 to 14 months of age, or in two stages with soft palate closure with or without lip plasty at 6 months of age, and hard palate closure at about 2 years of age. In the mixed dentition at 7 to 11 years of age, the residual cleft in the alveolar ridge is closed by a cancellous bone graft, harvested either from the iliac crest or the tibia.

The objective of Swedish healthcare quality registries is to ensure good quality of treatment for the patient, by enabling comparison and open reporting of results between different counties and hospitals. This is deemed as a necessity in order to promote quality control, research and improvement of treatment [2]. The Swedish Association of Local Authorities and Regions handles all certified national quality registries via six centres of registers [2].

The Swedish CLP registry may be seen as part of a global trend of assuring quality of CLP care. For example, the American Cleft Palate-Craniofacial Association [3] has stated the need for long-term follow-up by CLP teams to ensure the quality of cleft care. Since 2000, children born with CL/P in the United Kingdom (England, Wales and Northern Ireland) are registered in the CRANE Database [4], and in Norway, a quality registry for CLP started in 2011 [5]. In both North America [6] and France [7] projects are underway, aiming for standardized reporting of outcomes related to the treatment of CLP, enabling comparison between CLP centres.

Variables in a quality registry should be limited, well defined, easy to measure, reliable and allow for validation [8]. To achieve this, the variables and data of the CLP registry are continuously evaluated, and revisions of the variables are made when needed. The major part of the developmental work is carried out by the board of the registry, consisting of two professionals from each CLP centre, representing nurses, orthodontists, plastic surgeons and speech-language pathologists. In 2019, two patient representatives were included on the board of executives. All users of the registry regularly attend calibration meetings for the various professions.

Since 2016, data on treatment results from all six CLP centres have been reported annually [9]. The first review of data revealed both administrative problems and methodological measurement discrepancies, which were then solved. In the annual report of 2017, open comparisons of surgical data, and aggregated data on treatment results at 5 years of age were presented for the first time. The reliability of the registered orthodontic and speech data was assessed and evaluated to ensure a national consensus of the variables. In the annual report of 2018, open comparisons of all treatment outcomes were presented. From 2019, the results of quality indicators, based on data of orthodontics and speech, are published continuously on the Internet [10].

Efforts have been made to enable international inter-centre comparisons [11–16]. For example, an international multidisciplinary working group within the framework of the International Consortium for Health Outcomes Measurements (ICHOM) has presented a minimal standard set of outcome measures for cleft care [16]. In the Swedish CLP registry, we do not follow these recommendations unreservedly. However, with time, the design of the CLP registry and the guidelines of the ICHOM working group have become more similar regarding the amount and type of outcome measures that should be included in a CLP registry.

In order for data in a quality register to be reliable, the recorded data must be as complete as possible. The coverage rate for the individuals registered and the reporting degree for intervention and treatment outcomes should be as high as possible. The main objective was to investigate the coverage and reporting degree of the Swedish CLP registry, and to describe the design of the registry and discuss questions of reliability and validity of the data included.

## Methods

### Legal approval

Management of the recorded data follows Swedish law and Swedish implementation of the EU Data Protection Directive 95/46/EC [17, 18]. Personal data may be processed in a national or regional quality registry if the individual or caregivers approve. Information is given orally and in writing before registration. Written consent is not mandatory by law.

### Certification

The Swedish Association of Local Authorities and Regions has a three-level certification scale for the registries, with level 1 being the highest [2]. The Swedish CLP registry has reached level 2, which requires coverage higher than 60%, inclusion of patient-reported outcomes, online feedback to the users of the registry and open reporting of quality indicators. The registry should also be used for research. To achieve level 1, coverage should be higher than 85%. Information about the results directed to patients should be available on the web, and improved results in healthcare should be presented [2].

### IT solution

The CLP registry is connected to Centre of Registers South, which uses a web-based application (3C) for registration, storage and analysis, with encrypted data recording [19]. The system includes an advanced permissions module with strong authentication. To assure quality, statistical calculations are performed for all activities. The 3C server is located in a security-classified data centre and continuous back-up is executed [19].

### Recorded patients

Except for individuals with submucosal cleft palate or rare facial clefts, such as median cleft lip, all individuals in Sweden born from 1999 onwards with CL/P may be included. It is agreed that all children born from 2009 onwards should be recorded. A total of 2072 children born between 2009 and 2018 have been included.

### Recorded data

For all patients, data on treatment centre, civic registration number, date of birth, last name, first name, sex and date of first patient contact are registered.

#### Baseline data

Baseline data are primarily recorded at first visit/patient contact. Data registered at baseline are presented in Table 1. The receiving centre records patients who move between centres.

**Table 1:** Data recorded at baseline

Data recorded at baseline (information on Robin sequence, syndrome or other diagnosed deformity may be altered/supplemented later)	
Born in Sweden or not	
Adopted or not	
Birth weight	
Family history of clefts	
Pre-surgical orthopaedics: tape, plate, nasal alar elevator, nasoalveolar moulding or other	
Cleft morphology: right or left cleft extension in nasal floor, lip, alveolus, primary palate, hard and soft palate	
Primary ICD-10 code [20]	
Secondary ICD-10 [20] code if applicable	
Robin sequence, syndrome, or other diagnosed deformity	

Baseline data is principally based on examinations by pediatricians, ENT doctors and plastic surgeons. The plastic surgeons regularly calibrate themselves in terms of diagnostics and coding in the registry. If the cleft is suspected to be part of a syndrome, a geneticist also examines the child. At 5 years of age, a control of the baseline data takes place to ensure that correct data are registered.

#### Surgical data

Surgical data are recorded continuously. Information on cleft-related surgery performed abroad is also recorded based on anamnestic data. All operations are coded according to the Swedish National Board of Health and Welfare’s classification of health intervention [21]. The procedures are recorded as primary or secondary surgery, and by which anatomical structure the surgery was related to. If combined surgery, the procedure used is recorded with a major or minor surgical code. Data on duration of surgery and hospital stay, complications (i.e., bleeding, infection, rupture) and use of antibiotics (prophylaxis, postoperatively or both) are also recorded.

#### Orthodontic data

Orthodontic data are recorded for individuals with cleft soft and hard palate, unilateral CLP and bilateral CLP, at 5, 10, 16 and 19 years of age, and for individuals with cleft lip and alveolus at 16 years of age. In cases of orthognathic surgery, the orthodontic forms are filled in 1 year postoperatively. Data on occlusion and teeth are based on dental casts.

Because of substantial discrepancies among centres related to the orthodontic assessment procedure, two calibration meetings were performed, in 2016 and 2017. At the first meeting, the orthodontists agreed on revision of the included variables and standardization of the assessment procedure. Also, the included orthodontic variables were modified, in order to use validated indices with a worldwide spread [22, 23], enabling comparisons with other centres. Further, the orthodontists agreed to include a variable of ‘participation not possible’ to be able to separate missing, incomplete or lost data.

Dental arch relationship is assessed according to the Modified Huddart Bodenham index (MHB) [22], describing anterior and lateral cross-bites at 5, 10, 19 years of age as well as 1 year after orthognathic surgery. Additionally, for patients born with unilateral CLP, the dental arch relationship is assessed using the Atack index at 5 years [23] and the GOSLON yardstick from 10 years [24]. At 10 and 19 years of age as well as 1 year post orthognathic surgery, the cephalometric angles SNA, SNB, ML/NL and NAPg are assessed from lateral cephalograms, describing facial growth. At 10 years of age, agenesis of permanent teeth in the maxilla and the mandible is assessed from panoramic radiographs. Until now, orthodontic results up to 10 years of age have been recorded. For individuals who have been treated with bone-grafting, we will also record bone level in the cleft area according to modified Bergland index [25] at 16 years of age.

At the second calibration meeting, the orthodontists also performed blinded re-assessments of dental casts and photos. Agreement between data in the registry and re-assessments was investigated, and the registry data were found to be reliable for the chosen indices [26].

#### Speech data

The speech form is used for individuals with cleft soft palate, cleft soft and hard palate, unilateral CLP and bilateral CLP, at 5, 10, 16 and 19 years of age. Additional variables that may influence speech are recorded, such as residual cleft alveolus, fistula, diagnosed language impairment, diagnosed developmental disorder, diagnosed hearing impairment and services received from speech-language pathologists (i.e., routines and reviews, treatment and number of visits since last registration). Data are recorded on nasoendoscopy and/or videofluoroscopy performed for assessment of the velopharyngeal function, and if speech has been documented with video recording, audio recording or both at the speech assessment.

Speech data are based on perceptual assessment from standardized audio recordings, according to the assessment procedure of The Swedish Articulation and Nasality Test (SVANTE) [27]. Perceived velopharyngeal competence, i.e., an overall assessment of hypernasality, audible nasal air leakage and weak articulation, is rated on a three-point scale with the scale values ‘competent/sufficient’, ‘marginally incompetent/insufficient’ and ‘incompetent/insufficient’ [27]. Perceived velopharyngeal competence has previously been validated [28], and in a study by Brunnegård et al. [29], the reliability of data on perceived velopharyngeal competence in the CLP registry was good to excellent. The percentage of consonants correct and percentage of non-oral speech errors are recorded [27], based on phonetic transcriptions according to the International Phonetic Alphabet [30], for the 59 target consonants in SVANTE [27]. Percentage measures of consonant production have been widely used in cleft palate speech research [27]. In the study by Brunnegård et al. [29], agreement between judges for the percentage of correct consonants was excellent, with a single-measures intra-class coefficient of 0.85. Further, reliability of data on the percentage of non-oral errors in the CL/P registry was excellent in the study by Brunnegård et al., and in a previous study [31].

Since 2016, results from the Intelligibility in Context Scale [32], a patient/caregiver-reported validated measure of functional intelligibility, are registered at the ages of 5 and 10 years. In addition, since 2019 a separate form for registration at 18 months of age is used. Information on whether oral stops and anterior stops are established, and the size of consonant inventory are recorded.

### Investigation of coverage and reporting degree

Coverage degree for children born 2009 to 2018 in the CLP registry was assessed by comparing the number of individuals in the CLP registry with the number of individuals with a cleft diagnosis according to ICD-10 [20] in the Central patient registry, run by the Swedish National Board of Health and Welfare.

In Sweden, all surgical intervention is coded according to a classification system of the Swedish National Board of Health and Welfare. The reporting degree of surgical intervention in the CLP registry for children born 2009 to 2018 was assessed by comparing the number of cleft-related surgical intervention codes in the CLP registry with the number of surgical intervention codes in the Central patient registry for each individual during 2009 to 2018.

The number of orthodontic and speech registrations at 5 years of age for children born 2009–2013 was compared with the number of children registered at baseline. Children born abroad, children who had moved abroad, children who had been transferred between treatment centres and deceased children were excluded when calculating the reporting degree for orthodontics and speech.

## Results

### Coverage degree

**Fig. 1:**
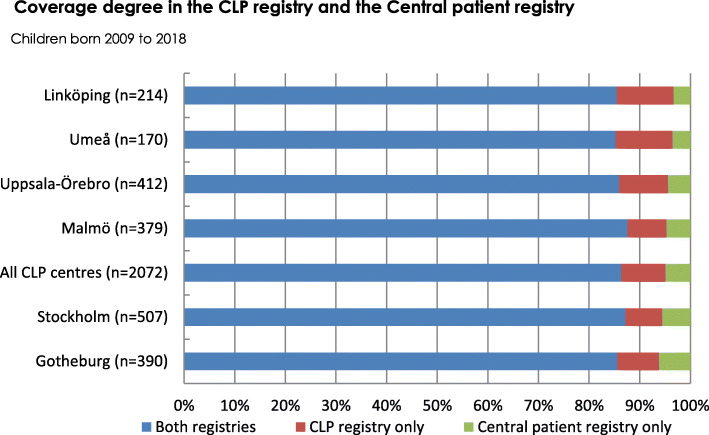
Coverage degree in the Swedish cleft lip and palate (CLP) registry

The average coverage degree was 95.1% and it was above 90% at all centres (Fig. 1).

### Reporting degree

**Fig. 2:**
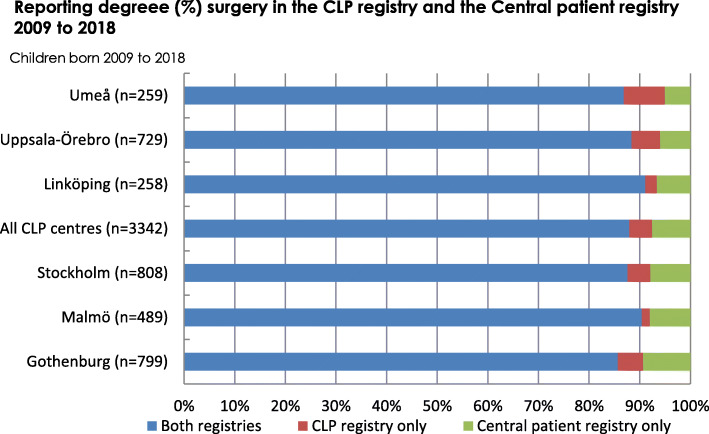
Average reporting degree for cleft-related surgeries in the Swedish cleft lip and palate (CLP) registry

The average proportion of reported cleft-related surgeries in the CLP registry was 92.4% and it was above 90% at all centres (Fig. 2).

**Fig. 3:**
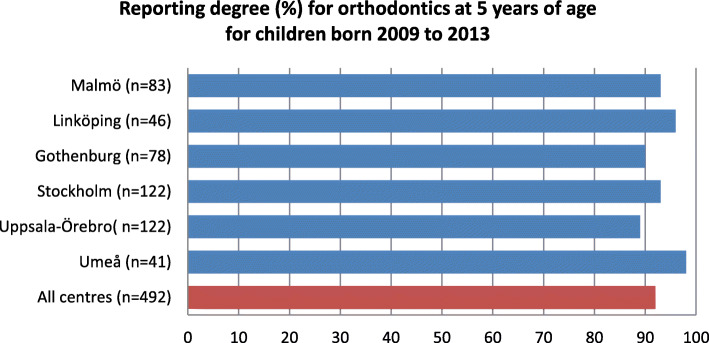
Reporting degree for orthodontics in the Swedish cleft lip and palate registry

The average reporting degree for orthodontics at 5 years of age was 92% and varied from 89 to 98% at the different CLP centres (Fig. 3).

**Fig. 4:**
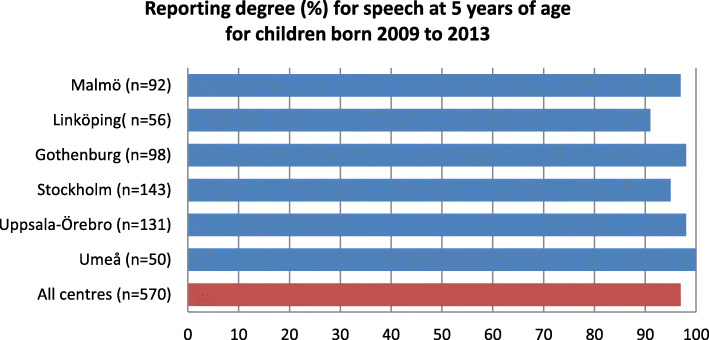
Reporting degree for speech in the Swedish cleft lip and palate registry

The average reporting degree for speech at 5 years of age was 97.5% and varied from 91 to 100% at the different centres (Fig. 4).

## Discussion

The objective of Swedish healthcare quality registries is to ensure good quality of treatment and to allow for comparison and open reporting of results in order to improve treatment [2]. To enable this, the coverage of children recorded and the reporting degree of the included data must be high. Also, the variables included in the registry must be valid and reliable. Outcomes of CLP treatment, based on the Swedish CLP registry, are published annually for open comparison. To our knowledge, today only two other quality registries in the world, the CRANE Database in the UK [4] and the Norwegian CLP registry [5], publish treatment results annually for open comparison between CLP centres.

### Degree of coverage and reporting

A high degree of coverage and reporting for all data included in healthcare quality registries is a prerequisite for the validity and usefulness of the data. When compared to the Swedish Central patient registry, the average coverage degree for children recorded in the Swedish CLP registry, born 2009 to 2018, was 95.1%. Possible reasons for patients being registered in the Central patient registry and not the CLP registry are patients declining participation in the CLP registry, and other caregivers who are not CLP specialists wrongly using a cleft diagnosis. In the CRANE Database, children with CL/P are recorded at some point between referral to the CLP team and the first primary surgery [4]. However, coverage degree has not been reported. All families with newborns with CL/P should be approached for consent. Of families with children born in 2017, the decision to provide or decline consent was made by 64% of the families, and 98.7% of them provided consent [4]. In the Norwegian CLP registry, children with CL/P are recorded in conjunction with the first cleft-related surgery, and in the annual report of 2018 coverage degree was reported to be 88.5% [5].

The average reporting degree for surgery in the CLP registry was 92.4% for 2009 to 2018. For orthodontics, the average reporting degree in the Swedish CLP registry, at 5 years of age for children born 2009 to 2013, was 92%, and the average reporting degree for speech was above 97%. For healthcare quality registries in Sweden to achieve certification level 2, coverage must be above 60%, and for level 1 it must be above 85% [2]. Further, in large inter-centre studies, a loss of 15% of the patients may be expected and accepted [13]. Considering this, the degree of coverage and reporting in the Swedish CLP registry is good.

Over recent years we have worked purposefully in order to increase the reporting degree for all forms. The Swedish healthcare system has no uniform system for medical records and does not offer the possibility to automate input to the national CLP registry. This means that all data collection must be done by hand at each CLP centre. The collection of data during ongoing routine medical care may be perceived as an additional imposed time-consuming work task. To provide an incentive for continuous recording of data, health professionals must perceive the registry as an aid in the development of healthcare [8]. Data in healthcare registries need to be recorded in real time to be up to date and allow for continuous excerpts. To achieve this, the activities need to be supported by the CLP centres’ principal organizers, and registry activities should be explicitly included in the mandate of the professionals at the CLP centres. In order to give the caregivers regular feedback on their work and promote high reporting degree of orthodontic data as well as speech data, we regularly publish the number of completed registrations at 5 years of age in relation to the expected number of completed registrations on the registry’s website [9].

### Data on cleft diagnoses, additional deformities and/or syndromes, and surgery

Over the years, the need for improved definitions and user guidelines for several of the variables included in the Swedish CLP registry has been revealed. For example, there were indications of non-uniform use of primary and, if existent, secondary cleft diagnosis. Since the extent of the cleft may influence treatment results [33], it is highly important to ensure uniform use of the ICD-10 codes [20] so that children with different cleft diagnoses can be studied in separate groups. Discrepancies were detected in the use of the classifications ‘primary surgery’ and ‘secondary surgery’ and in the numbers of children with diagnosed additional deformities and/or syndromes at the different CLP centres. In order to harmonize the coding of surgical procedures, cleft diagnoses and the criteria for diagnosing additional deformities and/or syndromes, calibration among surgeons is performed regularly.

### Orthodontic data

Agreement between orthodontic data in the Swedish CLP registry and re-assessments has been found to be reliable for the chosen indices [26], and the reporting degree to be good. It was considered possible to use orthodontic data for open comparison. As an indicator of quality, *Proportion of children with normal frontal relation* (based on MHB anterior score [22]) was chosen. A normal frontal relation is defined as an MHB anterior score ≥ − 2, on a scale from + 2 to − 6. This indicator of quality has been published for open comparison of data from the CL/P registry on the Internet [10].

The Atack index [23], included for 5-year-olds with unilateral CLP in the Swedish CLP registry, is also included in the CRANE Database [4]. Thus, it may be possible to compare data between registries in the future.

### Speech data

Since the reporting degree and reliability of speech data in the Swedish CLP registry has proven to be good, speech data allow for open comparisons. Based on the speech variable percentage of non-oral errors included in the CLP registry, Brunnegård et al. [29] developed a quality indicator, *Proportion of children without non-oral errors*. To have a margin of error, children were allowed to have up to 5% non-oral errors without being counted as having non-oral errors, and this quality indicator was found to be reliable. The second speech-related quality indicator, *Proportion of children with competent or marginally incompetent velopharyngeal function,* based on the variable perceived velopharyngeal competence, was also found to be reliable. These two quality indicators have been published for open comparison of data from the CLP registry online [10]. The third quality indicator, *Proportion of children with > 86% correct consonants*, needs further evaluation before publication on the Internet [29].

The speech data in the Swedish CLP registry are based on assessment with SVANTE [27]. The data in the Norwegian CLP registry are based on an adaption of SVANTE, and furthermore, the Norwegian CLP registry has incorporated the three above-mentioned speech-related quality indicators [5]. In the CRANE Database, speech data are based on The Cleft Audit Protocol for Speech – Augmented (CAPS-A) [34]. Although SVANTE and CAPS-A are based on the same principles of analysing speech, the speech outcome measures are not comparable.

### Patient-reported outcomes

To achieve a comprehensive evaluation of CLP care, patient-reported outcomes also need to be included [16]. A parent-reported outcome of intelligibility, the Intelligibility in Context Scale [32], is used at the ages of 5 and 10 years. In the near future, the registry will be supplemented with a patient-reported experience measure, Experience of Service Questionnaire (ESQ) [35], on how the patients experience the given care. ESQ is also used in the CRANE Database [4]. In the future, we will include patient-reported outcome measures in the Swedish CLP registry, targeting how children/adolescents experience the outcome of the care received. In this work, we will collaborate with psychologists and delegates from the patient group.

### Limitations

The Swedish CLP registry is now used for regular review of treatment results. However, retrospective analyses for comparisons of treatment results, which may influence the planning of healthcare, should ideally be based on new analyses of archived raw data by several blinded judges from different centres, in order to be able to determine the reliability of results. Thus, if differences among treatment centres are detected when reviewing registered data, it is important to go back and examine the raw data collected before any conclusions are made retrospectively. We also want to emphasize that to be able to draw conclusions about final treatment results, the patients must be fully grown and treatment completed. Today we only have valid data up to 10 years of age.

## Conclusions

In healthcare registries, the degree of coverage and reporting needs to be high, the variables included should be limited and checked for reliability, and the professionals must calibrate themselves regularly in order to achieve valid and reliable data. These criteria have been achieved in the Swedish CLP registry, which now allows for open comparisons of outcomes of treatment between CLP centres in order to develop and improve CLP care. However, we want to emphasize that if differences among treatment centres are detected, the raw data collected should be evaluated before any conclusions are made retrospectively.

## Data Availability

To access the data in the Swedish CLP registry, approval from an Ethics Board and Region Skåne is required (https://vardgivare.skane.se/kompetens-utveckling/forskning-inom-region-skane/utlamnande-av-patientdata-samradkvb/). Lists of included variables and results published in annual reports may be retrieved in Swedish from the website of the CLP registry: http://lkg-registret.se/.

## Abbreviations

CAPS-A: The Cleft Audit Protocol for Speech – Augmented
CLP: Cleft lip and palate
CL/P: Cleft lip and/or palate
CRANE: Cleft Registry and Audit Network
ESQ: Experience of Service Questionnaire
ICD-10: International Classification of Disease 10th Revision
ICHOM: International Consortium for Health Outcomes Measurements
MHB: Modified Huddart Bodenham index
ML/NL: Mandibula line/nasal line (measures the vertical inclination between the maxilla and the mandible)
NAPg: Nasion A-point – pogonion (measures skeletal facial convexity)
SNA: Sella nasion A-point angle (measures the sagittal position of the maxilla in relation to the anterior cranial base)
SNB: Sella nasion B-point angle (measures the sagittal position of the mandible in relation to the anterior cranial base)
SVANTE: The Swedish Articulation and Nasality Test
